# Similar Slow Component of Oxygen Uptake and Ventilatory Efficiency between an Aerobic Dance Session on an Air Dissipation Platform and a Constant-Load Treadmill Test in Healthy Women

**DOI:** 10.3390/biology11111646

**Published:** 2022-11-10

**Authors:** Alessandra Moreira-Reis, José Luis Maté-Muñoz, Juan Hernández-Lougedo, Pablo García-Fernández, Juan Ramón Heredia-Elvar, Eulogio Pleguezuelos, Teresa Carbonell, Norma Alva, Manuel Vicente Garnacho-Castaño

**Affiliations:** 1 Department of Cell Biology, Physiology and Immunology, Faculty of Biology, University of Barcelona, 08028 Barcelona, Spain; 2 Department of Radiology, Rehabilitation and Physiotherapy, Complutense University of Madrid, 28040 Madrid, Spain; 3 Department of Physiotherapy, Faculty of Health Sciences, Camilo José Cela University, 28692 Madrid, Spain; 4 Instituto de Investigación Sanitaria del Hospital Clínico San Carlos (IdISSC), 28040 Madrid, Spain; 5 Department of Physical Activity and Sports Science, Alfonso X El Sabio University, 28691 Madrid, Spain; 6 Physical Medicine and Rehabilitation Department, Hospital de Mataró, 08304 Barcelona, Spain; 7 Campus Docent Sant Joan de Déu, University of Barcelona, 08830 Sant Boi de Llobregat, Spain

**Keywords:** oxygen uptake kinetics, VE·VCO_2_^−1^ slope, cardiorespiratory responses, blood lactate, rating of perceived exertion, cardiopulmonary exercise test

## Abstract

**Simple Summary:**

The evaluation of the kinetics of oxygen uptake is considered an essential practice to analyze the effect of exercise intensity, mainly in endurance exercise. This study aimed to compare the slow component of oxygen uptake, ventilatory efficiency, blood lactate concentration, and the rating of perceived exertion between an aerobic dance session on an air dissipation platform and a constant-load treadmill test. Seventeen young adult and healthy women (aged 23.5 ± 2.2 years) completed three evaluation sessions. In session 1, an incremental test to exhaustion was completed. In sessions 2 and 3, the participants were randomly assigned to the aerobic dance session on an air dissipation platform or to a treadmill test at a constant-load corresponding to the first ventilatory threshold. No significant differences were found between the constant-load treadmill test and aerobic dance session on an air dissipation platform regarding the slow component of oxygen uptake, ventilatory efficiency, and the rating of perceived exertion. Higher blood lactate concentrations were observed in the aerobic dance session on an air dissipation platform than in the constant-load treadmill test. In conclusion, two different exercise modalities elicited similar slow components of oxygen uptake, ventilatory efficiency, and ratings of perceived exertion, even though the blood lactate concentrations were different.

**Abstract:**

There is a lack of evidence about the slow component of oxygen consumption ($\dot{V}O_{2\mathrm{sc}}$) and ventilatory efficiency (slope VE·VCO_2_^−1^) during an aerobic dance (AD) session on an air dissipation platform (ADP) despite the key role played in endurance exercises. This research was designed to assess $\dot{V}O_{2\mathrm{sc}}$, ventilatory efficiency, and blood lactate concentration by comparing two exercise modes: AD session on an ADP versus treadmill test at a constant-load intensity of the first ventilatory threshold (VT1). In the first session, an incremental treadmill test was completed. In sessions 2 and 3, the participants were randomly assigned to the AD session on an ADP or to a treadmill constant-load test at VT1 intensity to determine their cardioventilatory responses. In addition, their blood lactate levels and ratings of perceived exertion (RPE, CR-10) were evaluated. No significant differences were found between the constant-load treadmill test and AD session on an ADP with respect to $\dot{V}O_{2\mathrm{sc}}$, VE VCO_2_^−1^ slope, and RPE (*p* > 0.05). Higher blood lactate concentrations were observed in an AD session on an ADP than in a constant-load treadmill test at 10 min (*p* = 0.003) and 20 min (*p* < 0.001). The two different exercise modalities showed similar $\dot{V}O_{2\mathrm{sc}}$ and VE·VCO_2_^−1^ slope, even though the blood lactate concentrations were different.

## 1. Introduction

The evaluation of the kinetics of oxygen uptake ($\dot{V}O_{2}$) is considered an essential practice to analyze the effect of exercise intensity on so-called endurance exercises [1] and resistance exercises [2] in several populations [3,4,5,6]. Specifically, the pulmonary $\dot{V}O_{2}$ has a tendency to raise beyond 3 min during any constant work rate exercise involving sustained lactic acidosis, and above that of the primary component initiated at exercise onset. This ventilatory response generated in the kinetics of $\dot{V}O_{2}$ is known as the slow component of $\dot{V}O_{2}$($\dot{V}O_{2\mathrm{sc}}$) [7].

Cardioventilatory function could be altered by various parameters that condition the acute response of the $\dot{V}O_{2\mathrm{sc}}$, such as the load intensity, the lactate threshold (LT), and the ventilatory threshold (VT) [2,8]. At exercise intensities below LT and VT, a steady state of $\dot{V}O_{2}$ has been observed without increasing the $\dot{V}O_{2\mathrm{sc}}$ and blood lactate concentration. At exercise intensities above the LT or VT, an increase in the $\dot{V}O_{2\mathrm{sc}}$ has been verified depending on the increase in the exercise intensity and the blood lactate concentration. At a load intensity of the LT or the first ventilatory threshold (VT1), a steady state of both the $\dot{V}O_{2}$ and the blood lactate concentration has been observed, as well as a slight-moderate increase in the $\dot{V}O_{2\mathrm{sc}}$, both in endurance exercises and resistance exercises [2,9,10].

Cardiorespiratory performance is also frequently assessed by means of ventilatory efficiency. The relationship between ventilation (VE) and perfusion in the lungs is a key respiratory physiological mechanism to determine ventilatory efficiency in healthy people, athletes [11,12], and in those with respiratory diseases [13]. The VE/perfusion mismatch decreases the efficiency of pulmonary gas exchange, causing a state of hyperpnea and dyspnea that affects ventilatory performance [14]. The slope between the linear relationship of VE and carbon dioxide (VE·VCO_2_^−1^ slope) has been frequently used as a prognostic marker of ventilatory efficiency during an incremental test up to the anaerobic or ventilatory threshold [15,16], the ventilatory compensation point [11], in constant-load endurance and resistance exercise tests, and in resistance exercises of moderate and very high intensities [17,18].

As with the $\dot{V}O_{2\mathrm{sc}}$, exercise intensity also modulates the ventilatory efficiency response [18]. As exercise intensity increases, ventilation is increased to remove CO_2_ and maintain homeostatic control of pH [12], arterial hypoxemia [19], and lactic acidosis [20], thereby conditioning ventilatory efficiency [18]. At a constant-load intensity of the LT, similar to VT1, it has been proposed that both endurance exercises and resistance exercises maintain an efficient VE·VCO_2_^−1^ slope in a predominantly aerobic metabolism with a low blood lactate concentration (~2.7 mmol·L^−1^) [17]. It has been suggested that both the $\dot{V}O_{2\mathrm{sc}}$ and ventilatory efficiency could be conditioned, at least in part, by the type of exercise [17,21]. The $\dot{V}O_{2\mathrm{sc}}$ is higher in cycling compared to running exercises [21], and it even increases to a greater extent in the half-squat exercise compared to the cycle ergometer test at LT intensity [2]. However, the VE·VCO_2_^−1^ slope has generated controversy due to the lack of further studies in this regard with which to draw more accurate conclusions. Some studies showed that the VE·VCO_2_^−1^ slope is dependent on the mode of exercise [22], whereas others discard this hypothesis [11].

Aerobic dance (AD) classes are a very popular type of exercise worldwide, especially among women. AD is a choreographed activity of moderate to vigorous intensity accompanied by music and perceived as enjoyable [23,24]. Parameters such as $\dot{V}O_{2}$, heart rate (HR), and blood lactate concentration have been frequently used to determine the exercise intensity during a normal AD session [25]. Recent contributions from our research group have determined that the execution surface in AD activities can influence cardiorespiratory and metabolic responses. Unstable surfaces with a higher elastic component, such as an air dissipation platform (ADP), could increase the blood lactate concentration (~6 mmol·L^−1^) and cardioventilatory response during an AD session higher than on hard surfaces [26]. This increase in acute cardioventilatory and metabolic responses after the ADP session can be due to a decline in the damping-induced impact forces and an enhance in instability and contact times. From a physiological perspective, greater muscular tissue stimulation is expected in agonist and antagonist muscles [27]. This amplified activation can increase muscle work, causing a higher demand of the anaerobic metabolic pathway compared to a hard surface [28].

Other studies have shown that the type of AD conditions the exercise intensity to a greater degree compared to various intensities on a treadmill [29]. Therefore, it seems that the efficacy of AD exercises depends on the type of dance, the exercise intensity, and the surface of execution.

A steady state of $\dot{V}O_{2}$ and blood lactate concentration, as well as an adequate ventilatory efficiency with a low blood lactate concentration (~2.7 mmol·L^−1^), has been observed at a load intensity of the LT or the VT1 in predominantly aerobic metabolism (2, 9, 10, and 17). However, higher cardioventilatory responses and blood lactate concentrations (~6 mmol·L^−1^) have been verified in an AD session on an ADP compared to a hard surface [26]. Given the differences observed between several exercise modes, the treadmill, and various types of AD, it is plausible to propose that AD session on an ADP could condition the kinetics of $\dot{V}O_{2}$ by increasing the $\dot{V}O_{2\mathrm{sc}}$ and decreasing ventilatory efficiency to a greater extent than a treadmill test at an intensity of VT1 (~3 mmol·L^−1^), in which aerobic metabolism predominates. However, to the best of our knowledge, the $\dot{V}O_{2\mathrm{sc}}$ and ventilatory efficiency during an AD session on an ADP have not been studied to date. This knowledge would allow us to investigate other variables to interpret how the intensity of exercise is modulated in an AD session on an ADP.

The purpose of this study was to assess the $\dot{V}O_{2\mathrm{sc}}$, the VE·VCO_2_^−1^ slope, and blood lactate concentration by comparing a constant speed treadmill test at VT1 loading intensity with an AD test on an ADP in healthy young adult women. We hypothesize that an AD test on an ADP raises the $\dot{V}O_{2\mathrm{sc}}$ and the VE·VCO_2_^−1^ slope to a greater degree than a treadmill trial at VT1 loading intensity

## 2. Materials and Methods

### 2.1. Study Design

This is a cross sectional, comparative study. The participants completed three assessment sessions over 21 days in the exercise physiology laboratory. In the first test session, an incremental treadmill trial to exhaustion was completed to assess peak and VT1 cardiorespiratory responses. At the second testing session, which took place one week later, participants were randomly assigned to either the AD test on an ADP or a treadmill trial with a constant speed corresponding to the intensity of VT1. One week later, session 3 was held, in which the participants performed the pending protocol that was not conducted in session 2, and under the same conditions. The objective in sessions 2 and 3 was to determine the cardioventilatory responses for the subsequent analysis of the $\dot{V}O_{2\mathrm{sc}}$ and VE·VCO_2_^−1^ slope. Blood lactate concentration and rating of perceived exertion (RPE, CR-10) produced in sessions 2 and 3 were evaluated. The participants refrained from any type of physical exertion 24 h before starting the evaluation sessions.

### 2.2. Participants

Seventeen healthy women (23.5 ± 2.2 years, 58.6 ± 6.8 kg, 162.6 ± 0.1 cm, and 22.4 ± 2.5 body mass index) who were lightly physically active or moderate a maximum of 2 to 3 times per week completed the three test session. Participants were familiarized with the experimental procedures (AD and treadmill protocols). The following exclusion criteria were established: (1) any cardiovascular, metabolic, neurological, pulmonary, or orthopedic disorders that could limit exercise performance; (2) being an elite athlete; (3) tobacco or alcohol intake; and (4) the use of any performance-enhancing medication, drug, or supplement. The participants were informed of all experimental tests and signed an informed consent form. The study protocol received the approval of the University Ethics Committee and was carried out in accordance with the principles of the Declaration of Helsinki.

### 2.3. Treadmill Test

Before the start of the evaluation sessions on a treadmill (TechnoGym, Runrace 1400HC, Forlí, Italy), a 5-min warm-up was carried out at a speed of 5–6 km h^−1^, followed by 5 min of dynamic joint mobility exercises. Next, the incremental treadmill protocol was started at an initial speed of 5.5 km h^−1^ (1% slope), which was increased by 0.5 km h^−1^ every 30 s, until exhaustion. The constant speed test was performed at VT1 load intensity for 20 min.

### 2.4. Aerobic Dance Session

Each participant performed an AD session on an ADP. The ADP consists of a platform of one meter in diameter and 20 cm high, which rests on an elastomer that contains air at atmospheric pressure and allows for the entry and exit of air through holes. The air stored in the area produces a rebound effect, lessening the impact and rising instability during movements on the platform. First, a 5-min warm-up on the ADP was performed, followed by 5 minutes of dynamic joint mobility exercises. Subsequently, the main part of the AD test was carried out on an ADP, which lasted 20 min. The AD test was implemented by an experienced trainer to be executed at a constant light to moderate intensity as in a previous study [26].

### 2.5. Cardiorespiratory Record

In the two sessions on a treadmill and in the AD session on an ADP, respiratory exchange data were recorded using a breath-to-breath open-circuit gas analyzer (Vmax spectra 29, Sensormedics Corp., Yorba Linda, CA, USA), which had been previously calibrated. The following variables were recorded: $\dot{V}O_{2}$, VE, carbon dioxide production (VCO_2_), ventilatory oxygen equivalent (VE·$\dot{V}O_{2}$^−1^), ventilatory equivalent of carbon dioxide (VE·VCO_2_^−1^), respiratory exchange rate (RER), partial pressure of oxygen at expiration (PetO_2_), and partial pressure of carbon dioxide at expiration (PetCO_2_). HR was controlled every 5 sec by telemetry (RS-800CX, Polar Electro OY, Helsinki, Finland). VT1 was defined as the workload (speed) at which increases occurred in both VE∙$\dot{V}O_{2}$^−1^ and PetO_2_, with no concomitant increase in VE∙VCO_2_^−1^ [30].

During the constant-load test at an intensity of VT1 on a treadmill and the AD session on an ADP, the kinetics of pulmonary $\dot{V}O_{2}$ were evaluated. Pulmonary $\dot{V}O_{2}$ data were recorded during the 2 min prior to the start of the constant test (baseline state). Baseline $\dot{V}O_{2}$ ($\dot{V}O_{2B}$) was considered to mean the last 60 s before the start of the test. The fundamental kinetics (Phase II) of $\dot{V}O_{2}$ were determined using the criteria described above and were adjusted to a mono-exponential function, $\dot{V}O_{2}$(t) = $\dot{V}O_{2B}$ + ∆$\dot{V}O_{2\mathrm{FP}}$ · (1 − e^−(t−TR)/τ^) [31], where $\dot{V}O_{2}$(t) is the value of pulmonary $\dot{V}O_{2}$ at any time *t* of the kinetics of $\dot{V}O_{2}$, $\dot{V}O_{2B}$ is the value of basal $\dot{V}O_{2}$, ∆$\dot{V}O_{2\mathrm{FP}}$ is the increase in $\dot{V}O_{2}$ above the reference values and determines the range of the fundamental phase, and τ is the time constant of the fundamental phase. TR is the delay time. The exponential region of each participant was adjusted individually [32]. To determine the $\dot{V}O_{2\mathrm{sc}}$ (Phase III), the data of pulmonary $\dot{V}O_{2}$ were recorded. Finally, the $\dot{V}O_{2\mathrm{sc}}$ was determined in each participant: ∆$\dot{V}O_{2\mathrm{sc}}$ = $\dot{V}O_{2\mathrm{peak}}$ − ($\dot{V}O_{2B}$ + ∆$\dot{V}O_{2\mathrm{FP}}$) [32].

### 2.6. Metabolic Fatigue and Rating of Perceived Exertion

Metabolic fatigue was assessed by means of a transportable lactate apparatus (Lactate Pro LT-1710, Arkray Factory Inc., KDK Corporation, Siga, Japan). For this purpose, blood lactate concentration was quantified in the capillary of the index finger at rest, at 10 min and at the end of the test. The RPE was controlled applying the Borg scale (CR-10) [33] and was assessed in accordance with blood lactate measurements.

### 2.7. Statistical Analysis

The Shapiro-Wilk test was applied for testing multivariate normality. To apply the statistical treatment, the data were presented as mean, percentages and confidence intervals (95% CI). 

A Student’s *t*-test for paired samples was utilized to verify statistically significant changes between both experimental sessions (ADP vs. treadmill test). A two-way analysis of variance (ANOVA) for repeated measures was completed to confirm significant differences between an AD session on an ADP and a constant-load treadmill test with respect to blood lactate concentration and RPE. When appropriate, a Bonferroni post hoc adjustment for multiple comparisons was implemented. The magnitude of the response was calculated using the partial eta-squared (ηp^2^). Sample size was estimated with α = 0.05 (5% probability of type I error) and 1 − β = 0.80 (power 80%). The statistical power (SP) was also calculated using G*Power 3. The Pearson product–moment correlation coefficients were calculated to determine significant relationships between the VE and the VCO_2_. SPSS Statistics software package version 25.0 for Mackintosh (SPSS, Chicago, IL, USA) was used for statistical procedures. Statistical significance was set at *p* < 0.05.

## 3. Results

The data related to the incremental treadmill test are indicated in Table 1.

**Figure 1:**
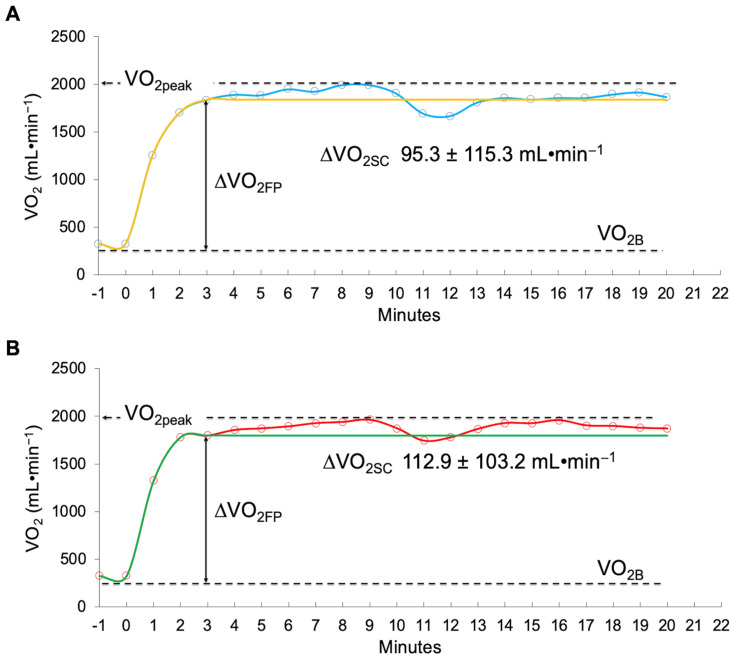
Slow component of oxygen uptake ($\dot{V}O_{2\mathrm{sc}}$): (**A**) Aerobic dance on an air dissipation platform (blue line: $\dot{V}O_{2\mathrm{sc}}$ of participants_;_ yellow line: $\dot{V}O_{2\mathrm{sc}}$ expected at VT_1_) (**B**) Constant speed treadmill test at VT1 intensity (red line: $\dot{V}O_{2\mathrm{sc}}$ of participants; green line: $\dot{V}O_{2\mathrm{sc}}$ expected at VT_1_). Abbreviations: ∆VO_2FP_ = increase in oxygen uptake in the fundamental phase; VO_2B_ = baseline oxygen consumption.

**Figure 2:**
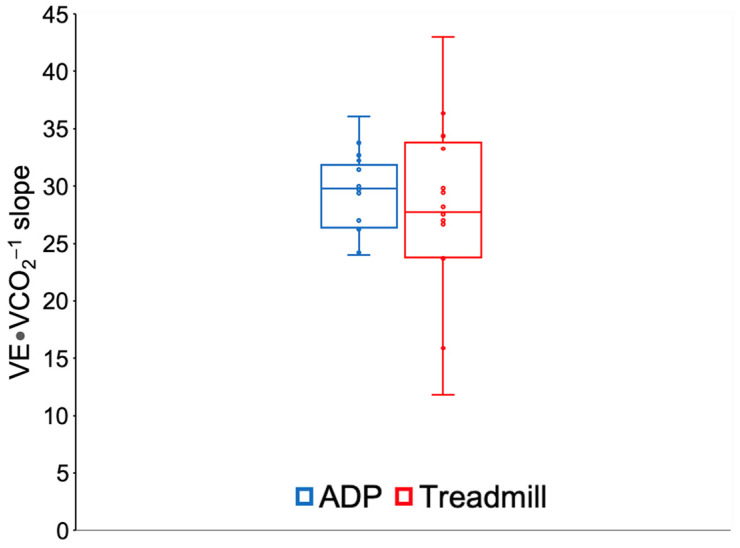
Ventilatory efficiency (VE·VCO_2_^−1^ slope) during aerobic dance (AD) exercise on an air dissipation platform (ADP) and constant-load treadmill test at VT1 intensity.

No significant differences were found between the constant-load treadmill test at VT1 intensity and the AD session on an ADP with respect to the $\dot{V}O_{2\mathrm{sc}}$ (*p* = 0.642; Figure 1) and the VE·VCO_2_^−1^ slope (*p* = 0.520; Figure 2).

Excellent correlations were verified between the VE and VCO_2_ in the constant-load treadmill test (r = 0.93; *p* < 0.001) and in the AD session on an ADP (r = 0.83; *p* < 0.001).

The differences between the constant-load treadmill test at VT1 intensity and the AD session on an ADP concerning the blood lactate concentration and RPE are shown in Table 2.

Regarding the blood lactate concentration, a significant interaction effect (mode·time) was observed (*p* = 0.001, ηp^2^ = 0.6, SP = 0.9). Significant time and mode effects were detected (*p* < 0.001, ηp^2^ = 0.9, SP = 1.0; *p* < 0.001, ηp^2^ = 0.6, SP = 0.9, respectively). The Bonferroni post hoc adjustment determined significantly higher blood lactate concentrations in the AD session on an ADP than in the constant-load treadmill test at 10 min (*p* = 0.003) and 20 min (*p* < 0.001). Concerning the RPE, no significant interaction and mode effects were verified (*p* > 0.05). A significant time effect was found (*p* = 0.034, ηp^2^ = 0.3, SP = 0.6).

## 4. Discussion

Contrary to our study hypothesis, the main finding was that although the AD session on an ADP induced higher blood lactate concentrations than the constant-load treadmill test, both protocols (treadmill versus ADP) elicited similar responses in the $\dot{V}O_{2\mathrm{sc}}$, ventilatory efficiency, and RPE in the healthy young adult women.

The results observed with respect to the $\dot{V}O_{2\mathrm{sc}}$ (absolute values: 95.3 mL in 20 min; relative values: 4.68 mL·min^−1^) were slightly lower (not significant) than those verified in the treadmill test (absolute values: 113 mL in 20 min; relative values: 5.65 mL·min^−1^). In previous studies with samples of similar characteristics, we found slightly higher values in African American women (absolute values: 120 mL in approximately 9 min; relative values: 13.33 mL·min^−1^) and much higher values in Caucasian women (absolute values: 170 mL in ~8 min; relative values: 21.25 mL·min^−1^) at an intensity of 25% above the gas exchange threshold in a cycle ergometer test [34]. It is likely that the differences observed can be attributed to the exercise intensity applied in each study and to the fact that the cycle ergometer tends to raise the $\dot{V}O_{2\mathrm{sc}}$ to a greater extent than a treadmill at the same relative intensity [21].

Another study found increases in $\dot{V}O_{2}$ of 599 mL·min^−1^ in women with lupus erythematosus and 540 mL·min^−1^ in healthy sedentary women during a 6-minute walk test on a treadmill at an intensity of 3 MET [35]. Despite being a lower intensity test compared to our study, the $\dot{V}O_{2}$ rose to a greater extent beyond the third minute to the sixth than during our twenty-minute test, indicating that cardiorespiratory fitness could also be another fundamental element that conditions the $\dot{V}O_{2\mathrm{sc}}$ [36]. We did not find a relationship between cardiorespiratory fitness and the $\dot{V}O_{2\mathrm{sc}}$ reported in other studies [37].

The physiological mechanisms that influence $\dot{V}O_{2\mathrm{sc}}$ are diverse as well as uncertain. The kinetics of $\dot{V}O_{2}$ showed a similar steady state from the third minute in the AD session and in the treadmill test, justifying the similarity of the exercise intensity in both tests, which were corroborated by a very similar $\dot{V}O_{2\mathrm{sc}}$. In theory, the blood lactate concentration should not increase in response to a clearly aerobic metabolism in both experimental conditions. However, the blood lactate concentration increased significantly after the AD session on an ADP compared to the treadmill test, evidencing a transition to increased anaerobic metabolism [38]. In the AD session on an ADP, there was a paradoxical result, since the increase in the blood lactate concentration would indicate an exercise intensity above the LT or anaerobic threshold [39], which in theory should increase the $\dot{V}O_{2\mathrm{sc}}$. The fact that the $\dot{V}O_{2\mathrm{sc}}$ did not increase could be due, at least in part, to the fact that there was a significant degree of energy contribution from anaerobic sources.

It is likely that the supply of O_2_ to active skeletal muscle was deficient, since the intensity of muscle contraction was intense enough to cut off arterial and venous flow, causing other predominantly anaerobic metabolic sources to be activated [40]. In this regard, it is possible that an elastic surface such as an ADP contributed to the increased blood lactate levels. Previous research has shown that elastic or softer surfaces increase blood lactate concentration to a greater extent than hard surfaces, while maintaining a stationary and similar $\dot{V}O_{2}$ [26,41]. Instability and contact times increase during an AD session on an ADP. The impact forces induced by the damping are reduced, causing greater muscle activation in the agonist and antagonist muscles [27]. This amplified muscle activation could increase muscle work and potentiate a higher demand from the anaerobic sources compared to a hard surface [28]. In addition, a significant part of the energy from the anaerobic sources cannot be quantified by gas exchange [42], which explains, to a certain extent, why an increase in the $\dot{V}O_{2}$ and $\dot{V}O_{2\mathrm{sc}}$ amplitude was not observed. It is likely that the increased lactate production during an AD session would be beneficial to supplying the anaerobic source demands induced by an ADP. More studies will be needed to substantiate such claims.

The increased anaerobic environment produced by the increased blood lactate concentration in the AD session on an ADP would be expected to induce an increase in the VE·VCO_2_^−1^ slope [18]. The increase in exercise intensity induces an increase in the blood lactate levels, CO_2_ levels_,_ and the number of hydrogen ions [H^+^]. The accumulation of lactate and [H^+^] is mainly due to intracellular glycolysis [43,44], and they are both released into the extracellular fluid [45,46]. In theory, the ventilatory efficiency would decrease as a result of the increasing ventilation required to eliminate the CO_2_ produced in order to maintain pH homeostasis, causing a mismatch in the ventilation–perfusion relationship. However, no changes were noted in terms of ventilatory efficiency between both exercise modalities; thus, this mismatch in the ventilation–perfusion relationship did not occur, which is typical as the exercise intensity increases [18]. This confirms that the exercise intensity in both modalities was not high enough to cause this mismatch in the ventilation–perfusion ratio.

Comparing them with other studies with healthy adult women, we found similar values (VE·VCO_2_^−1^ slope of 28.7) during an incremental protocol on a treadmill [47]. In another study, slightly lower VE·VCO_2_^−1^ values (24.1) were reported during a cycle ergometer test in healthy adult women [11]. In strenuous high-intensity exercise with blood lactate concentrations close to 18 mmol·L^−1^, the VE·VCO_2_^−1^ slope could exceed values of 40 [18].

From a mechanistic perspective, the VE·VCO_2_^−1^ slope data (Figure 2) showed different variabilities between the treadmill test and the AD session on an ADP. The analysis of the differences between both modes of exercise accounted for the different variances between both groups. In this case, no homogeneity of variances was identified between both groups (homoscedasticity).

Few studies have compared the VE·VCO_2_^−1^ slopes between different exercises modalities. Our findings showed that two different exercise modalities with similar responses in $\dot{V}O_{2}$ and the $\dot{V}O_{2\mathrm{sc}}$ and different metabolic responses had similar ventilatory efficiency values. Other studies found no difference between treadmill and cycle ergometer tests, although both exercise modalities showed a lower VE·VCO_2_^−1^ slope compared to a robotics-assisted tilt table [48]. There appears to be a trend towards a similar VE·VCO_2_^−1^ slope between different exercise modalities at the same relative intensity [12].

A similar RPE was observed in both exercise modalities, which indicates the enjoyment that the AD session can produce [24].

Several of this study’s limitations should be considered. AD is a complex exercise modality for assessing the $\dot{V}O_{2\mathrm{sc}}$, since maintaining constant $\dot{V}O_{2}$ kinetics, unlike the constant-load test (treadmill), depends on the intensity, which is always determined by the AD monitor. Although the kinetics of $\dot{V}O_{2}$ were similar between both exercise modes, the movements performed in the AD session on an ADP were different from those observed in the treadmill test. This different methodology could have influenced the final results.

## 5. Conclusions

Our findings indicated that the AD session on an ADP and the treadmill test at a constant-load intensity of VT1 elicited similar $\dot{V}O_{2\mathrm{sc}}$, VE·VCO_2_^−1^ slope, and RPE values in healthy young adult women, despite the fact that the AD session on an ADP induced higher blood lactate concentrations than the treadmill test.

## Figures and Tables

**Table 1:** Cardioventilatory data achieved during the incremental treadmill test.

Variable	N	Minimum	Maximum	Mean	SD
HR (beats∙min^−1^)	17	173.0	198.0	187.2	7.4
Absolute peak $\dot{V}O_{2}$ (L∙min^−1^)	17	1.7	3.6	2.4	0.5
Relative peak $\dot{V}O_{2}$ (mL∙kg^−1^∙min^−1^)	17	30.1	54.8	41.4	7.5
Peak VCO_2_ (L∙min^−1^)	17	2.0	3.9	2.9	0.5
Peak RER	17	1.1	1.4	1.2	0.1
Peak VE (L∙min^−1^)	17	54.7	109.7	84.7	17.0
Peak VE·$\dot{V}O_{2}$^−1^	17	29.0	47.0	36,5	5.1
Peak VE·VCO_2_^−1^	17	28.0	36.0	32.5	2.2
Peak Speed (km∙h^−1^)	17	9.5	15.0	12.2	1.7
HR at VT1 (beats∙min^−1^)	17	127.0	174.0	150.1	13.1
Absolute $\dot{V}O_{2}$ at VT1 (L∙min^−1^)	17	0.6	2.5	1.5	0.5
Relative $\dot{V}O_{2}$ at VT1 (mL∙kg^−1^∙min^−1^)	17	11.1	37.1	25.4	7.2
VCO_2_ at VT1 (L∙min^−1^)	17	0.5	2.2	1.3	0.4
RER at VT1	17	0.7	1.0	0.9	0.1
VE at VT1 (L∙min^−1^)	17	17.9	59.6	37.7	10.4
VE·$\dot{V}O_{2}$ ^−1^ at VT1	16	21.0	34.0	25.4	3.6
VE·VCO_2_^−1^ at VT1	16	24.0	34.0	28.8	3.2
Speed at VT1 (km∙h^−1^)	17	5.0	9.0	7.1	1.2

Abbreviations used: HR = heart rate; RER = respiratory exchange rate; SD = standard deviation; VCO_2_ = carbon dioxide production; VE = minute ventilation; VE·VCO_2_^−1^ = ventilatory equivalent of carbon dioxide; VE·$\dot{V}O_{2}$^−1^ = ventilatory oxygen equivalent; $\dot{V}O_{2}$ = oxygen uptake; VT1 = first ventilatory threshold.

**Table 2:** Differences in blood lactate concentration and RPE between an AD session on an ADP and a constant-load treadmill test at VT1 intensity.

	ADP (95% CI)			Treadmill (95% CI)			*p* ^1^	*p* ^2^	*p* ^3^
	Rest	10 min	20 min	Rest	10 min	20 min	ES/SP	ES/SP	ES/SP
Lactate (mmol·L^−1^)	1.5 *	5.9 ^Ψ^	6.5 ^#^	1.5 *	3.8 ^δ^	2.9	**<0.001**	**<0.001**	**<0.001**
	(1.2–1.7)	(4.6–7.4)	(5.2–7.7)	(1.3–1.7)	(3.0–4.6)	(2.3–3.7)	(0.5–0.9)	(0.8–1.0)	(0.6–0.9)
RPE		10.3	10.7		10.8	11.6	0.300	**0.034**	0.318
		(9.8–10.8)	(10.0–11.4)		(9.6–12.0)	(9.9–13.3)	(0.1–0.2)	(0.3–0.6)	(0.1–0.2)

Abbreviations used: AD—aerobic dance; ADP—air dissipation platform; ES—effect size; RPE—ratio of perceived effort; SP—statistical power; VT1—first ventilatory threshold. *p* ^1^ Significant differences for exercise mode × time interaction effect. *p* ^2^ Significant differences for time effect. *p* ^3^ Significant differences for exercise mode effect. Data are provided as mean and 95% confidence intervals (95% CI). Bonferroni post hoc adjustment for multiple comparisons: ^Ψ^ Significant differences compared to 10 min treadmill (*p* = 0.003). ^#^ Significant differences compared to 20 min treadmill test (*p* < 0.001). * Significant differences compared to 10 min and 20 min tests (*p* < 0.001, AD session on an ADP; *p* ≤ 0.002, treadmill). ^δ^ Significant differences compared to 20 min treadmill test (*p* = 0.016).

## Data Availability

Not applicable.

## References

[1] Lucía A., Hoyos J., Santalla A., Pérez M., Chicharro J.L. (2002). Kinetics of VO(2) in professional cyclists. Med. Sci. Sports Exerc..

[2] Garnacho-Castaño M.V., Albesa-Albiol L., Serra-Payá N., Bataller M.G., Felíu-Ruano R., Cano L.G., Cobo E.P., Maté-Muñoz J.L. (2019). The Slow Component of Oxygen Uptake and Efficiency in Resistance Exercises: A Comparison With Endurance Exercises. Front. Physiol..

[3] Fawkner S.G., Armstrong N., Potter C.R., Welsman J.R. (2002). Oxygen uptake kinetics in children and adults after the onset of moderate-intensity exercise. J. Sports Sci..

[4] George M.A., McLay K.M., Doyle-Baker P.K., Reimer R.A., Murias J.M. (2018). Fitness Level and Not Aging per se, Determines the Oxygen Uptake Kinetics Response. Front. Physiol..

[5] Kemps H.M., Schep G., Zonderland M.L., Thijssen E.J., de Vries W.R., Wessels B., Doevendans P.A., Wijn P.F. (2010). Are oxygen uptake kinetics in chronic heart failure limited by oxygen delivery or oxygen utilization?. Int. J. Cardiol..

[6] Regensteiner J.G., Bauer T.A., Reusch J.E.B., Brandenburg S.L., Sippel J.M., Vogelsong A.M., Smith S., Wolfel E.E., Eckel R.H., Hiatt W.R. (1998). Abnormal oxygen uptake kinetic responses in women with type II diabetes mellitus. J. Appl. Physiol..

[7] Gaesser G.A., Poole D.C. (1996). The slow component of oxygen uptake kinetics in humans. Exerc. Sport Sci. Rev..

[8] Burnley M., Jones A.M. (2007). Oxygen uptake kinetics as a determinant of sports performance. Eur. J. Sport Sci..

[9] Garnacho-Castaño M.V., Palau-Salvà G., Cuenca E., Muñoz-González A., García-Fernández P., del Carmen Lozano-Estevan M.D.C., Veiga-Herreros P., Maté-Muñoz J.L., Domínguez R. (2018). Effects of a single dose of beetroot juice on cycling time trial performance at ventilatory thresholds intensity in male triathletes. J. Int. Soc. Sports Nutr..

[10] Garnacho-Castaño M.V., Dominguez R., Maté-Muñoz J.L. (2015). Understanding the Meaning of Lactate Threshold in Resistance Exercises. Endoscopy.

[11] Sun X.-G., Hansen J.E., Garatachea N., Storer T.W., Wasserman K. (2002). Ventilatory Efficiency during Exercise in Healthy Subjects. Am. J. Respir. Crit. Care Med..

[12] Brown S.J., Raman A., Schlader Z., Stannard S.R. (2013). Ventilatory efficiency in juvenile elite cyclists. J. Sci. Med. Sport.

[13] Arena R., Myers J., Guazzi M. (2007). The clinical and research applications of aerobic capacity and ventilatory efficiency in heart failure: An evidence-based review. Heart Fail. Rev..

[14] Reindl I., Kleber R.X. (1996). Exertional hyperpnea in patients with chronic heart failure is a reversible cause of exercise intolerance. Basic Res. Cardiol..

[15] Brown S.J., Brown J.A. (2009). Heart Rate Variability and Ventilatory Efficiency. Endoscopy.

[16] Baba R., Nagashima M., Goto M., Nagano Y., Yokota M., Tauchi N., Nishibata K. (1996). Oxygen uptake efficiency slope: A new index of cardiorespiratory functional reserve derived from the relation between oxygen uptake and minute ventilation during incremental exercise. J. Am. Coll. Cardiol..

[17] Albiol L.A., Paya N.S., Garnacho-Castaño M.A., Cano L.G., Cobo E.P., Maté-Muñoz J.L., Garnacho-Castaño M.V. (2019). Ventilatory efficiency during constant-load test at lactate threshold intensity: Endurance versus resistance exercises. PLoS ONE.

[18] Serra-Payá N., Garnacho-Castaño M., Sánchez-Nuño S., Albesa-Albiol L., Girabent-Farrés M., Arcone L.M., Fernández A., García-Fresneda A., Castizo-Olier J., Viñals X. (2021). The Relationship between Resistance Exercise Performance and Ventilatory Efficiency after Beetroot Juice Intake in Well-Trained Athletes. Nutrients.

[19] Romer L.M., Polkey M.I. (2008). Exercise-induced respiratory muscle fatigue: Implications for performance. J. Appl. Physiol..

[20] Whipp B.J. (1994). The The bionergetic and gas exchange basis of exercise testing. Clin. Chest Med..

[21] Billat V.L., Richard R., Binsse V.M., Koralsztein J.P., Haouzi P. (1998). The VO_2_ slow component for severe exercise depends on type of exercise and is not correlated with time to fatigue. J. Appl. Physiol..

[22] Davis J.A., Tyminski T.A., Soriano A.C., Dorado S., Costello K.B., Sorrentino K.M., Pham P.H. (2006). Exercise test mode dependency for ventilatory efficiency in women but not men. Clin. Physiol. Funct. Imaging.

[23] Edvardsen E., Ingjer F., Bø K. (2011). Fit Women Are Not Able to Use the Whole Aerobic Capacity During Aerobic Dance. J. Strength Cond. Res..

[24] Rockefeller K.A., Burke E.J. (1979). Psycho-physiological analysis of an aerobic dance programme for women. Br. J. Sports Med..

[25] De Angelis M., Vinciguerra G., Gasbarri A., Pacitti C. (1998). Oxygen uptake, heart rate and blood lactate concentration during a normal training session of an aerobic dance class. Eur. J. Appl. Physiol. Occup. Physiol..

[26] Moreira-Reis A., Maté-Muñoz J.L., Hernández-Lougedo J., García-Fernández P., Pleguezuelos-Cobo E., Carbonell T., Alva N., Garnacho-Castaño M.V. (2020). Cardiorespiratory, Metabolic and Muscular Responses during a Video-Recorded Aerobic Dance Session on an Air Dissipation Platform. Int. J. Environ. Res. Public Health.

[27] Behm D.G., Anderson K., Curnew R.S. (2002). Muscle force and activation under stable and unstable conditions. J. Strength Cond Res..

[28] Brito J., Krustrup P., Rebelo A. (2012). The influence of the playing surface on the exercise intensity of small-sided recreational soccer games. Hum. Mov. Sci..

[29] Rixon K.P., Rehor P.R., Bemben M.G. (2006). Analysis of the assessment of caloric expenditure in four modes of aerobic dance. J. Strength Cond. Res..

[30] Lucía A., Hoyos J., Pérez M., Chicharro J.L. (2000). Heart rate and performance parameters in elite cyclists: A longitudinal study. Med. Sci. Sports Exerc..

[31] Keir D.A., Benson A.P., Love L.K., Robertson T.C., Rossiter H.B., Kowalchuk J.M. (2016). Influence of muscle metabolic heterogeneity in determining the $\dot{V}O_{2p}$ kinetic response to ramp-incremental exercise. J. Appl. Physiol..

[32] Murgatroyd S.R., Ferguson C., Ward S.A., Whipp B.J., Rossiter H.B. (2011). Pulmonary O_2_ uptake kinetics as a determinant of high-intensity exercise tolerance in humans. J. Appl. Physiol..

[33] Borg G. (1970). Perceived exertion as an indicator of somatic stress. Scand. J. Rehabil. Med..

[34] Lai N., Tolentino-Silva F., Nasca M.M., Silva M.A., Gladden L.B., Cabrera M.E. (2011). Exercise intensity and oxygen uptake kinetics in African-American and Caucasian women. Eur. J. Appl. Physiol..

[35] Keyser R.E., Rus V., Mikdashi J.A., Handwerger B.S. (2010). Exploratory Study on Oxygen Consumption On-kinetics During Treadmill Walking in Women With Systemic Lupus Erythematosus. Arch. Phys. Med. Rehabilitation.

[36] Russell A., Wadley G., Snow R., Giacobino J.P., Muzzin P., Garnham A., Cameron-Smith D. (2002). Slow component of VO_2_ kinetics: The effect of training status, fibre type, UCP3 MRNA and citrate synthase activity. Int. J. Obes. Relat. Metab. Disord..

[37] Reis V.M., Guidetti L., Duarte J.A., Ascensão A., Silva A.J., E Sampaio J., Russell A.P., Baldari C. (2007). Slow component of VO_2_ during level and uphill treadmill running: Relationship to aerobic fitness in endurance runners. J. Sports Med. Phys. Fit..

[38] O’Connell J., Weir J., MacIntosh B. (2017). Blood lactate accumulation decreases during the slow component of oxygen uptake without a decrease in muscular efficiency. Eur. J. Appl. Physiol..

[39] Svedahl K., MacIntosh B.R. (2003). Anaerobic Threshold: The Concept and Methods of Measurement. Can. J. Appl. Physiol..

[40] Tamaki T., Uchiyama S., Tamura T., Nakano S. (1994). Changes in muscle oxygenation during weight-lifting exercise. Eur. J. Appl. Physiol. Occup. Physiol..

[41] Murias J.M., Lanatta D., Arcuri C.R., Laiño F.A. (2007). Metabolic and functional responses playing tennis on different surfaces. J. Strength Cond. Res..

[42] Garnacho-Castaño M.V., Albesa-Albiol L., Serra-Payá N., Bataller M.G., Cobo E.P., Cano L.G., Guodemar-Pérez J., Carbonell T., Domínguez R., Maté-Muñoz J.L. (2021). Oxygen Uptake Slow Component and the Efficiency of Resistance Exercises. J. Strength Cond. Res..

[43] Stickland M.K., Lindinger M.I., Olfert I.M., Heigenhauser G.J.F., Hopkins S.R. (2013). Pulmonary Gas Exchange and Acid-Base Balance During Exercise. Compr. Physiol..

[44] Lindinger M.I., McKelvie R.S., Heigenhauser G.J. (1995). K+ and Lac- distribution in humans during and after high-intensity exercise: Role in muscle fatigue attenuation?. J. Appl. Physiol..

[45] Gladden L.B. (2004). Lactate metabolism: A new paradigm for the third millennium. J. Physiol..

[46] Goodwin M.L., Harris J.E., Hernández A., Gladden L.B. (2007). Blood Lactate Measurements and Analysis during Exercise: A Guide for Clinicians. J. Diabetes Sci. Technol..

[47] Habedank D., Reindl I., Vietzke G., Bauer U., Sperfeld A., Wernecke K.D., Kleber F.X. (1998). Ventilatory efficiency and exercise tolerance in 101 healthy volunteers. Eur. J. Appl. Physiol. Occup. Physiol..

[48] Saengsuwan J., Nef T., Laubacher M., Hunt K.J. (2015). Submaximal cardiopulmonary thresholds on a robotics-assisted tilt table, a cycle and a treadmill: A comparative analysis. Biomed. Eng. Online.

